# ILD-GAP Combined with the Charlson Comorbidity Index Score (ILD-GAPC) as a Prognostic Prediction Model in Patients with Interstitial Lung Disease

**DOI:** 10.1155/2023/5088207

**Published:** 2023-02-08

**Authors:** Hiroaki Fujii, Yu Hara, Yusuke Saigusa, Yoichi Tagami, Kota Murohashi, Ryo Nagasawa, Ayako Aoki, Ami Izawa, Kenichi Seki, Keisuke Watanabe, Nobuyuki Horita, Nobuaki Kobayashi, Takeshi Kaneko

**Affiliations:** ^1^Department of Pulmonology, Yokohama City University Graduate School of Medicine, Yokohama, Japan; ^2^Department of Biostatistics, Yokohama City University Graduate School of Medicine, Yokohama, Japan

## Abstract

**Background:**

The ILD-GAP scoring system has been widely used to predict the prognosis of patients with interstitial lung disease (ILD). The ability of the ILD-GAP scoring system combined with the Charlson Comorbidity Index score (CCIS) (ILD-GAPC) to predict ILD prognosis was investigated.

**Methods:**

In ILD patients, including idiopathic pulmonary fibrosis (IPF), idiopathic nonspecific interstitial pneumonia (iNSIP), collagen vascular disease-related interstitial pneumonia (CVD-IP), chronic hypersensitivity pneumonitis (CHP), and unclassifiable ILD (UC-ILD), treated between April 2013 and April 2017, the relationships between baseline clinical parameters, including age, sex, CCIS, ILD diagnosis, pulmonary function test results, and disease outcomes, were retrospectively assessed, and the ability to predict prognosis was compared between the ILD-GAP and ILD-GAPC models, respectively.

**Results:**

A total of 185 patients (mean age, 71.9 years), all of whom underwent pulmonary function testing, including percentage predicted diffusion capacity for carbon monoxide, were assessed. ILD diagnosis consisted of IPF in 57 cases, iNSIP and CVD-IP in 117 cases, CHP in 6 cases, and UC-ILD in 5 cases. The ILD-GAPC provided a greater area under the receiver operating characteristic curve (0.758) for predicting 3-year ILD-related events than the ILD-GAP (0.721). In addition, log-rank tests showed that the Kaplan−Meier curves differed significantly among low, middle, and high ILD-GAPC scores (*P* < 0.001), unlike ILD-GAP scores (*P* = 0.083).

**Conclusions:**

The ILD-GAPC model could provide more accurate information for predicting prognosis in patients with ILD than the ILD-GAP model.

## 1. Background

Interstitial lung disease (ILD) is characterized by alveolar inflammation leading to progressive fibrosis. The clinical course and rate of progression of ILD are extremely variable among patients due to various radiological and pathological-morphological patterns, such as usual interstitial pneumonia (UIP), nonspecific interstitial pneumonia (NSIP), organizing pneumonia, respiratory bronchiolitis, desquamative interstitial pneumonia, diffuse alveolar damage, and their combinations [1]. An official statement of the American Thoracic Society, the European Respiratory Society, the Japanese Respiratory Society, and the Latin American Thoracic Association (ATS/ERS/JRS/ALAT) proposed various clinical parameters associated with an increased risk of mortality, such as clinical symptoms, pulmonary function, and the extent of UIP on high-resolution computed tomography (HRCT); however, clinical parameters for accurately predicting the prognosis of ILD have not been established [2].

To provide more accurate prognostic information in patients with ILD, various composite approaches have been reported using peripheral blood biomarkers and physiological and radiographic measurements [3–7]. Ley et al. proposed the GAP index as a mortality prediction model for idiopathic pulmonary fibrosis (IPF) patients, consisting of four parameters including gender (G), age (A), percent predicted forced vital capacity (%FVC), and diffusion capacity of carbon monoxide (%D_Lco_) (P) [3]. In addition, to predict mortality in major chronic ILD subtypes including IPF, idiopathic NSIP (iNSIP), collagen vascular disease-related interstitial pneumonia (CVD-IP), chronic hypersensitivity pneumonia (CHP), and unclassifiable ILD (UC-ILD), ILD-GAP has been reported to be useful [4]. Both GAP and ILD-GAP have been widely used in the clinical setting, but these mortality prediction models do not take into account the presence or severity of comorbidities, despite previous research showing that comorbidities, such as cardiovascular disease, arteriosclerosis, and cancer, affect the long-term prognosis of ILD [8, 9].

The present study retrospectively investigated the accuracy of predicting ILD prognosis using the ILD-GAP scoring system combined with the Charlson Comorbidity Index score (CCIS) (ILD-GAPC), which has been widely used as a prognostic indicator for patients with colorectal cancer, advanced nonsmall cell lung carcinoma, acute myocardial infarction, and so on [10–13].

## 2. Methods

### 2.1. Study Location and Enrolled Patients

**Figure 1:**
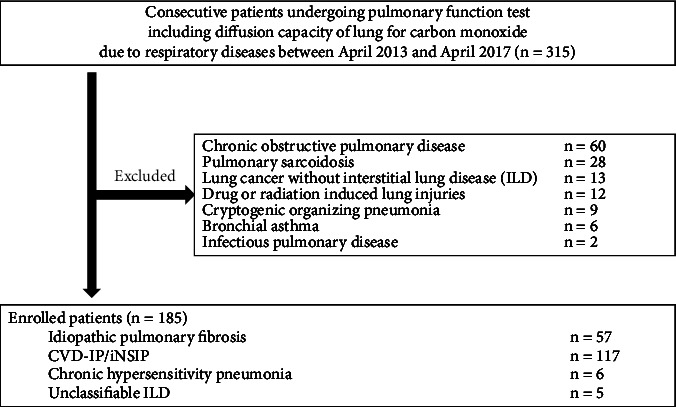
Flowchart of the participants' selection process.

This retrospective, observational study was performed using data from patients treated at Yokohama City University Hospital between April 2013 and April 2017. The medical records of all patients with ILD who met the following inclusion criteria were reviewed: patients with IPF, iNSIP, CVD-IP, CHP, and UC-ILD in a stable condition who were able to perform pulmonary function tests, including D_Lco_. ILD patients in a stable condition were defined as patients who had not experienced acute respiratory worsening such as an acute exacerbation (AE), infection, pulmonary embolism, pneumothorax, or pulmonary edema until a pulmonary function test [14]. As shown in Figure 1, pulmonary sarcoidosis, lung cancer with ILD at the time of enrollment, cryptogenic organizing pneumonia, drug or radiation-induced lung injuries, chronic obstructive pulmonary disease, bronchial asthma, and infectious pulmonary disease were excluded.

### 2.2. Data Collection

The relationships between baseline clinical parameters include age, sex, CCIS, ILD diagnosis, blood biomarker results, pulmonary function test results, and the disease outcome. CCIS is a summed score of 19 comorbidities weighted according to severity, which was developed to assess the risk of death from comorbidities and has been widely used as a prognostic indicator for patients with colorectal cancer, advanced nonsmall cell lung carcinoma, and acute myocardial infarction [10–13]. In recent years, large-scale cohort studies and clinical trials in fibrotic ILD have also been recognized as important factors affecting long-term prognosis, including mortality and acute exacerbation [14, 15]. The disease outcome included 3-year ILD-related events and 3-year all-cause mortality. Three-year ILD-related events mean ILD-related mortality such as respiratory failure and first AE after the pulmonary function test within 3 years. Three-year all-cause mortality includes respiratory events such as chronic respiratory failure due to ILD and nonrespiratory events such as extrapulmonary malignancy after the pulmonary function test within 3 years. For patients who did not die in our hospital, the disease outcomes were confirmed by telephone. In addition, only one patient (0.5%), who was transferred to another hospital for best supportive care due to severe deterioration of respiratory status, was lost to follow-up; therefore, the transfer date of that patient was selected as the decision date of the disease outcome.

### 2.3. Diagnosis of ILD

A diagnosis of idiopathic interstitial pneumonias (IIPs) was confirmed by physical findings, serological testing, findings from HRCT, and lung biopsy specimens, based on the official statement for IIP [1, 2]. Patients from whom a lung biopsy could not be obtained were diagnosed based on the radiological classification [1, 2]. The diagnosis of CVD-IP was confirmed by physical findings, serological testing, and HRCT findings consistent with ILD. CHP was diagnosed based on previously established criteria [16]. An AE of ILD was defined as: unexplained worsening of dyspnoea; hypoxemia or severely impaired gas exchange; new alveolar infiltrates on radiography; and absence of an alternative explanation such as infection, pulmonary embolism, pneumothorax, or pulmonary edema [17–19].

### 2.4. The Details of ILD-GAP and ILD-GAPC Classification

The ILD-GAP model was developed for application across all ILD subtypes, including iNSIP, CVD-IP, CHP, and UC-ILD to provide cause-specific survival estimates using a single risk prediction model compared to the original GAP model for IPF patients that accounted for better adjusted survival in these patients [4]. As shown in Table 1, the predictor variables considered in this model include gender, age, lung physiology variables (%FVC and %D_Lco_), and these ILD subtypes. The ILD-GAP score is calculated by combining the points assigned to these variables that is then divided into stages I (≤1 point), II (2, 3 points), III (4, 5 points), and IV (>5 points) or low score (≤1 point), moderate score (2, 3 points), and high score (≥4 points) that predict mortality risks at 1, 2, and 3 years. CCIS was scored as follows: (0-1: 0 points, 2-3: 1 point, ≥4: 2 points).The ILD-GAPC score is calculated by combining CCIS and the original ILD-GAP scores and then divided into low score (≤1 point), moderate score (2–3 points), and high score (≥4 points). The rationale for creating the ILD-GAPC model will be presented in the result section.

### 2.5. Statistical Analysis

Data were statistically analysed using JMP12 (SAS Institute, Cary, NC) and R software, version 3.5.1 (The R Foundation for Statistical Computing, Vienna, Austria), and are expressed as means ± standard deviation. Groups were compared using the chi-square test and Wilcoxon rank-sum tests. To determine the primary predictors of 3-year ILD-related events, including cause-specific mortality and the first AE, univariate analyses were performed. The predictive performance of the scoring systems was investigated using the areas under the time-dependent receiver operating characteristic curve (ROC) analysis (AUC), the concordance index (C-index), and Akaike's information criterion (AIC). When comparing 3-year ILD-related events and 3-year all-cause mortality among groups depending on the scoring system, Kaplan−Meier curves were used. Log-rank testing was also performed with strata based on the identified predictors. Values of *P* < 0.05 were considered significant.

## 3. Results

### 3.1. Patients' Characteristics

**Figure 2:**
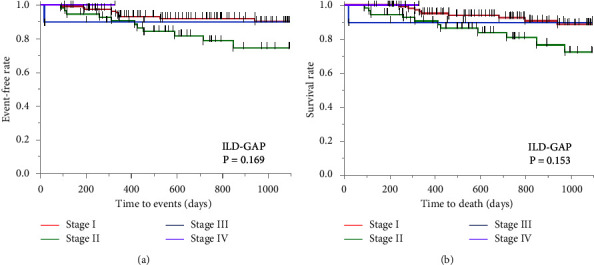
Kaplan−Meier curves according to the ILD-GAP classification. The enrolled ILD patients were divided into 4 stages according to the ILD-GAP classification (stage I, 117 cases; stage II, 57 cases; stage III, 10 cases; stage IV, 1 case). The Kaplan−Meier curves for predicting 3-year ILD related events (a) or 3-year all-cause mortality (b) proved to be not significant between 4 stages. ILD, interstitial lung disease; G/A/P, gender/age/physiology.

Table 2 shows the clinical characteristics of the 185 patients evaluated, including IPF in 57 cases, iNSIP and CVD-IP in 117 cases, CHP in 6 cases, and UC-ILD in 5 cases. CVD-IP included rheumatoid arthritis in 11 cases, antineutrophil cytoplasmic antibody-associated vasculitis in 5 cases, polymyositis/dermatomyositis in 7 cases, and Sjögren's syndrome in 8 cases. Especially in the IPF group, the incidence of males was the highest, and %D_Lco_ was the lowest. The ILD-GAP score between IPF and UC-ILD was similar and higher than the other ILDs. The antifibrotic agents were used in 10 patients, including 9 with IPF and 1 with iNSIP. Antiinflammatory agents, including corticosteroids or immunosuppressants, were used mainly in patients with CVD-IP or iNSIP. The enrolled ILD patients were divided into 4 stages according to the ILD-GAP model (stage I, 117 cases; stage II, 57 cases; stage III, 10 cases; stage IV, 1 case). As shown in Figure 2, the Kaplan−Meier curves for predicting 3-year ILD-related events (*P* = 0.169) or 3-year all-cause mortality (*P* = 0.153) proved to be not significant between the 4 stages.

### 3.2. Univariate Analysis of Primary Predictors of 3-Year ILD-Related Events

To determine the primary predictors of 3-year ILD-related events, univariate analysis was performed with the following parameters: age, sex, CCIS, diagnosis of ILD (IPF vs. non-IPF), ILD-GAP score, %FVC, and %D_Lco_ (Table 3). This showed that CCIS, the ILD-GAP score, and the FVC were significant predictors of 3-year ILD-related events.

### 3.3. Accuracy of Composite Scoring Models in Predicting 3-Year ILD-Related Events

It was hypothesized that the ILD-GAP model combined with CCIS (the ILD-GAPC model) is more accurate for predicting 3-year ILD-related events than the ILD-GAP model. Table 1 shows the details of ILD-GAPC scoring. Based on the previous research using nomogram analysis, CCIS was classified into three categories and scored as follows: (0-1: 0 points, 2-3: 1 point, ≥4: 2 points) [7]. Then, in the ILD-GAPC model, the score of CCIS was added to the ILD-GAP score. To investigate the accuracy of the ILD-GAP and ILD-GAPC models for 3-year ILD-related events, AUCs, C-index values, and AIC values for these models were calculated. All of the AUCs, C-index values, and AIC values were higher with the ILD-GAPC model than with the ILD-GAP model (Table 4).

### 3.4. Comparison of the Kaplan−Meier Curves between the ILD-GAP and ILD-GAPC Models

**Figure 3:**
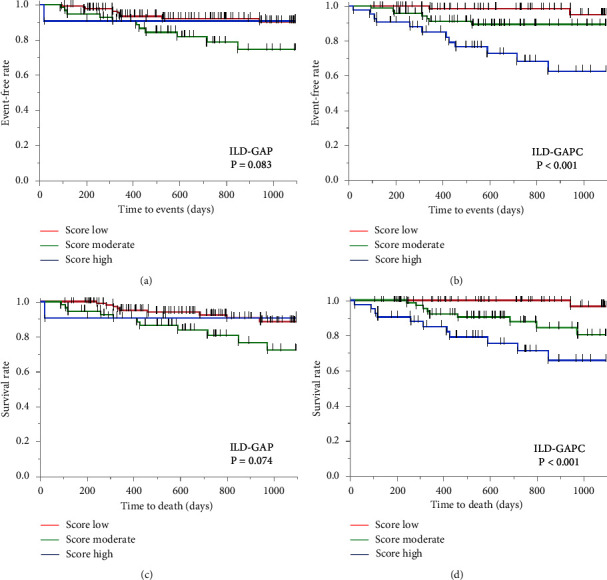
Comparison of the Kaplan–Meier curves between the ILD-GAP and ILD-GAPC models. (a) ILD-GAP *P*=0.083, and (b) ILD-GAPC *P* < 0.001, for 3-year ILD events. (c) ILD-GAP *P*=0.074, and (d) ILD-GAPC *P* < 0.001 for 3-year all-cause mortality.

As shown in Figure 2, using the classification from the original ILD-GAP model, the number of stage III and IV cases is very small. Considering the equality of cases in each group, we attempted to change the staging of the ILD-GAP and ILD-GAPC models. The Kaplan−Meier curves for 3-year ILD-related events were compared according to the ILD-GAP score (low score ≤1 point vs. moderate score 2, 3 points vs. high score ≥4 points), and the log-rank test showed that these groups did not differ significantly (Figure 3(a) (*P* = 0.083)). On the other hand, these curves were compared according to the ILD-GAPC score (low score ≤1 point vs. moderate score 2–3 points vs. high score ≥4 points), and the log-rank test showed that the Kaplan−Meier survival curves of these groups differed significantly (Figure 3(b) (*P* < 0.001)). Furthermore, log-rank tests showed that the Kaplan−Meier curves for 3-year all-cause mortality differed significantly among low, middle, and high ILD-GAPC scores (*P* < 0.001) (Figure 3(d)), unlike ILD-GAP scores (*P* = 0.074) (Figure 3(c)).

## 4. Discussion

Although the clinical course and rate of progression of ILD are extremely variable among patients, clinical parameters for accurately predicting the prognosis of ILD have not been established [1, 2]. From the viewpoint of clinical simplicity and versatility, various composite approaches such as GAP or ILD-GAP including age, sex, ILD diagnosis, and physiological measurements have been widely used to provide more accurate prognostic information in clinical settings [3, 4]. However, these mortality prediction models do not take into account the presence or severity of comorbidities. In the present study, the ILD-GAPC model was found to better predict 3-year ILD-related events and 3-year all-cause mortality than the ILD-GAP model.

FVC is widely used as a biomarker in patients with ILD for predicting prognosis or evaluating treatment efficacy [3, 4, 20–26]. Longitudinal variation of FVC is reported to be more reliable than baseline FVC, since baseline FVC may oversimplify the staging process because disease activity in patients with ILD does not always progress in a linear pattern [2, 26]. Actually, we demonstrated that the most influential prognostic factor was CCIS, not the baseline FVC. The CCIS, as a summed score of 19 comorbidities weighted according to severity, was developed to assess the risk of death from comorbidities and has been widely used as a prognostic indicator for patients with various diseases [11–13]. Also, in patients with ILD in both stable and AE conditions, the CCIS has been recently reported to be a prognostic indicator [6, 7, 14, 15, 27, 28]. Interestingly, in the present study, although the number of events was small, the ILD-GAPC model was shown to more sensitively predict ILD-related events, including first AE and mortality, rather than nonrespiratory mortality, for which only CVD-IP/iNSIP patients showed nonrespiratory death, and the calculation of the ILD-GAPC score is expected to be a prognostic biomarker specific to ILD (Supplement Figure 1). These suggest that the comorbidity itself has a direct impact on the progression of ILD, rather than simply coexisting with it. In order to prove this, it is necessary to analyze whether the definitive treatment of comorbidities improves the prognosis of ILD, however, it can be said that this is a future task.

ILD can be associated with a large number of comorbidities, such as lung cancer, diabetes mellitus, coronary artery disease, heart failure, pulmonary hypertension (PH), gastroesophageal reflux disease (GERD), and so on [29–34]. As shown above, the progression of comorbidities may be pathophysiologically linked to the progression of ILD itself; however, their prognostic impact and mechanism are not fully understood. Previous studies have revealed a high incidence of lung cancer in IPF (7% to 20%), though the true cumulative incidence of lung cancer after the diagnosis of IPF and its predictive factors at the initial diagnosis of IPF remain unknown. Various mechanisms such as endoplasmic reticulum stress, alterations of growth factors expression, oxidative stress, and large genetic and epigenetic variations, myofibroblast/mesenchymal transition, myofibroblast activation and proliferation can contribute to predispose the patient to develop IPF and lung cancer [29]. Diabetes mellitus is a systemic disorder characterized by a chronic hyperglycemic state that is associated with inflammation and oxidative stress, leading to interstitial fibrosis and alveolar capillary microangiopathy [30]. MicroRNAs (miRNAs) regulate gene expression at the posttranscriptional level, contributing to all major cellular processes, including oxidative stress and cell death. Several miRNAs have been reported to crosstalk with oxidative stress in both the cardiac and pulmonary systems [31]. Fibrogenic mediators such as transforming growth factor-*β* promote fibroblast migration, proliferation, and activation in the heart and lungs [32]. Mechanisms contributing to the development of PH in patients with IPF are complex, including hypoxia causing smooth muscle hypertrophy and collagen deposition in pulmonary arteries, the destruction and obstruction of pulmonary vasculature by the progression of pulmonary fibrosis, and vascular remodeling contributed by fibroblast growth factor and platelet-derived growth factor [33]. In patients with GERD and IPF, microaspiration of gastric material may play a fundamental role in the fibrotic transformation of pulmonary parenchyma, and IPF may favor GERD by increasing the negative intrathoracic pressure [34]. From these, the progression of ILD seems to crosstalk with other comorbidities, suggesting that comorbidities may contribute to ILD-related events even if they do not directly cause death. Thus, high CCIS not only indicates an increased risk of death from comorbidities but may also indicate a poorer prognosis for ILD itself.

The ILD-GAP model has been reported to accurately predict mortality in major chronic ILD subtypes such as IPF, iNSIP, CVD-IP, and CHP [4]. In the present study, the ILD-GAPC model was a better predictor of 3-year ILD-related events than the ILD-GAP model, though there was a significant correlation between these models (Supplement Table 1). Although all patients in the high ILD-GAP score group were included in the high ILD-GAPC score group, the patients in the moderate ILD-GAP score group were divided into the moderate and high ILD-GAPC score groups, and the patients in the low ILD-GAP score group were divided into the low and moderate ILD-GAPC score groups. The previously reported ILD-GAP model is a model for ILD patients with higher severity than in the present study, in fact, the enrolled patients in the original research on the ILD-GAP model had much lower %FVC and %D_Lco_ than those in the present study [4]. Based on the above, the ILD-GAP model is considered a prognosis prediction model for severe cases, while the ILD-GAPC model is considered a highly versatile model for patients with a wide range of severity from mild to severe.

Although the ILD-GAPC model might have been shown to be a useful scoring system to predict the incidence of AE or future mortality in patients with ILDs, there are several limitations in the present research. The number of enrolled patients was still small from a single institution. Especially, the clinical diagnoses of the patients enrolled with CHP or UC-ILD were much smaller than the others. Also, we used the CCIS as an assessment of the severity of ILD comorbidities, but we have not been able to compare it with other scoring model, such as the COPD specific comorbidity test (COTE) index, and so on [35, 36]. The reproducibility of the findings of this study needs to be confirmed through validation cohorts that increase the number of patients in the future. The majority of patients enrolled were not so severely ill that pulmonary function tests, including D_Lco_ could not be tolerated, which suggests a possible source of bias in the present research. Actually, the number of patients with a high ILD-GAP score is very small. The ILD-GAPC model is useful for examining the long-term prognosis of relatively mild cases, and future validation including more severe cases is also necessary, though only in the %FVC >75% (%FVC score: 0 point) populations, we found that ILD-GAPC better predicted the 3-year ILD-related events than ILD-GAP (Supplement Figure S1). A treatable traits approach has been proposed as a new paradigm for the management of chronic lung diseases such as chronic airway disease, bronchiectasis, and ILD [37–39]. Especially in ILD, from the recent reports of the clinical efficacy of anti-fibrotic agents, the detection and severity evaluation of lung fibrosis or inflammation as the treatable trait has become more important in considering therapeutic intervention [24, 25, 40]. As in the previous research, in which the CCIS proved to be an important prognostic indicator in patients with ILD, comorbidities such as lung cancer, cardiovascular disease, GERD, and PH have been reported to have prognostic impacts [8, 9]. Thus, not only lung involvements but also CCIS seemed to be important treatable traits for patients with ILD, though it is unclear whether treatment for these comorbidities will improve the prognosis of ILD patients (Supplement Figure 3).

## 5. Conclusions

We speculate that comorbidity itself has a direct impact on the progression of ILD, rather than simply coexisting with it. Also, a high CCIS not only indicates an increased risk of death from comorbidities but may also indicate a poorer prognosis for ILD itself. From the above, the ILD-GAPC model could provide more accurate information for predicting prognosis in patients with ILD than the ILD-GAP model.

## Figures and Tables

**Table 1:** ILD-GAP and ILD-GAPC models.

		ILD-GAP model	ILD-GAPC model
		Point	Point
ILD diagnosis	IPF/UC-ILD	0	0
	CVD-IP ± iNSIP ± CHP	−2	−2

Sex	Female	0	0
	Male	1	1

Age	≤60	0	0
	61–65	1	1
	>65	2	2

%FVC	>75	0	0
	50–75	1	1
	<50	2	2

%DLco	>55	0	0
	36–55	1	1
	≥35	2	2
	Cannot perform	3	3

CCIS	0–1		0
	2–3		1
	≤4		2

CCIS, Charlson comorbidity Index Score; CHP, chronic hypersensitivity pneumonitis; CVD-IP, collagen vascular disease-related interstitial pneumonia; GAP, gender/age/physiology; GAPC, gender/age/physiology/Charlson comorbidity index score; ILD, interstitial lung disease; iNSIP, idiopathic nonspecific interstitial pneumonia; IPF, idiopathic pulmonary fibrosis; %DLco, percentage predicted diffusion capacity of lung for carbon monoxide; %FVC, percentage predicted forced vital capacity; UC-ILD, unclassifiable interstitial lung disease.

**Table 2:** Patient's characteristics.

Characteristics	Overall cases	IPF	CVD-IP/iNSIP	CHP	UC-ILD	*P* value
Total number, *n* (%)	185 (100)	57 (30.8)	117 (63.2)	6 (3.2)	5 (2.7)	
Age (years)	71.9 ± 9.1	73.3 ± 7.2	71.4 ± 9.2	67.8 ± 21.3	72.4 ± 4.0	0.608
Male sex, *n* (%)	124 (67.0)	49 (86.0)	66 (56.4)	4 (66.7)	5 (100)	<0.001
CCIS	2.5 ± 2.1	2.6 ± 2.0	2.5 ± 2.2	1.5 ± 1.2	2.00.7	0.611
Blood biomarker						
KL-6 (U/mL)	867.7 ± 1112.2	748.3 ± 456.5	839.4 ± 1071.6	2692 ± 3265.3	405.2 ± 154.4	0.116
Pulmonary function tests						
%FVC	94.2 ± 18.8	93.5 ± 18.5	94.1 ± 19.2	92.8 ± 10.8	105 ± 19.6	0.408
%FEV1	90.8 ± 19.8	87.3 ± 19.2	92.1 ± 20.4	92.7 ± 8.7	97.3 ± 19.8	0.370
%DLco	92.9 ± 29.7	81.9 ± 26.2	97.6 ± 30.2	83.1 ± 17.6	118.9 ± 25.0	0.001
Emphysematous lesion, *n* (%)	105 (57)	49 (86)	56 (48)	0	0	<0.001
ILD-GAP score	1.4 ± 1.4	3.0 ± 0.9	0.6 ± 0.7	0.7 ± 0.5	3.2 ± 0.4	<0.001
Treatment						
Anti-fibrotic agents, *n* (%)	10 (5.4)	9 (15.8)	1 (0.9)	0 (0)	0 (0)	<0.001
Corticosteroid, *n* (%)	42 (22.7)	9 (15.8)	32 (27.4)	1 (1.6)	0 (0)	0.205
Immunosuppressant, *n* (%)	20 (10.8)	0 (0)	20 (17.1)	0 (0)	0 (0)	<0.001
Outcome						
Follow-up (days)	792 ± 479	757 ± 469	821 ± 495	815 ± 350	484 ± 211	0.402
3-yr ILD-related events, *n* (%)	21 (11.4)	10 (17.5)	10 (8.5)	0 (0)	1 (0.5)	0.238
3-yr all-cause mortality, *n* (%)	21 (11.4)	9 (15.8)	11 (9.4)	0 (0)	1 (0.5)	0.441
Respiratory causes	15	9	5	0	1	0.040
Nonrespiratory causes	6	0	6	0	0	0.308

3-yr ILD-related events include cause specific mortality due to ILD and first AE after pulmonary function test within 3 years. Therefore, we excluded the patients who did not have experienced an AE but died of non-respiratory causes. Abbreviations: AE, acute exacerbation; CHP, chronic hypersensitivity pneumonia; CCIS, Charlson comorbidity index score; CI, confidence interval; CVD-IP, collagen vascular disease-related interstitial pneumonia; GAP, gender/age/physiology; ILD, interstitial lung disease; IPF, idiopathic pulmonary fibrosis; KL-6, Krebs von den Lungen; %DLco, percentage predicted diffusion capacity of lung for carbon monoxide, %FVC, percentage predicted forced vital capacity; UC-ILD, unclassifiable interstitial lung disease.

**Table 3:** Univariate analysis of primary predictors of 3-year ILD-related events.

Variable	Hazard ratio	95% CI	*P*
Age	1.010	0.991–1.030	0.296
Sex (male)	1.218	0.472–3.140	0.680
CCIS	1.529	1.300–1.786	<0.001
Diagnosis of ILDs	2.154	0.914–5.077	0.085
ILD-GAP score	1.486	1.095–2.012	0.011
%FVC	0.975	0.952–0.998	0.036
%DLco	0.988	0.971–1.004	0.139

CCIS, Charlson comorbidity index score; G/A/P, gender/age/physiology; ILD, interstitial lung disease; %DLco, percentage predicted diffusion capacity of lung for carbon monoxide, %FVC, percentage predicted forced vital capacity.

**Table 4:** Predictability for ILD-related events in the ILD-GAP and ILD-GAPC models.

	Time dependent AUROC	C-index	AIC
ILD-GAP model	0.721	0.549	202.5
ILD-GAPC model	0.758	0.662	193.6

AIC, akaike's information criterion; AUROC, areas under the receiver operating characteristic curve; GAP, gender/age/physiology; GAPC, gender/age/physiology/Charlson comorbidity indec score; ILD, interstitial lung disease.

## Data Availability

The data used to support the findings of this study are available from the corresponding author upon request.

## Abbreviations

AE: Acute exacerbation
AIC: Akaike's information criterion
ATS/ERS/JRS/ALAT: American Thoracic Society/the European Respiratory Society/the Japanese Respiratory Society/the Latin American Thoracic Association
AUC: Areas under the time-dependent receiver operating characteristic curve
C-index: Concordance index
CCIS: Charlson comorbidity index score
CHP: Chronic hypersensitivity pneumonia
CVD-IP: Collagen vascular disease-related interstitial pneumonia
GAP: Gender/age/physiology
GERD: Gastroesophageal reflux disease
HRCT: High-resolution computed tomography
IIP: Idiopathic interstitial pneumonia
ILD: Interstitial lung disease
iNSIP: Idiopathic non-specific interstitial pneumonia
IP: Interstitial pneumonia
IPF: Idiopathic pulmonary fibrosis
LDH: Lactate dehydrogenase
%D_Lco_: Percentage predicted diffusion capacity of lung for carbon monoxide
%FVC: Percentage predicted forced vital capacity
PH: Pulmonary hypertension
ROC: Receiver operating characteristic curve
UC-ILD: Unclassifiable interstitial lung disease
UIP: Usual interstitial pneumonia.

## Supplementary Materials

Supplementary Materials Supplement Figure 1: The ILD-GAPC score according to ILD-related events or nonrespiratory mortality. Figure legends: the ILD-GAPC score with ILD-related events was significantly higher than those without (3.6 ± 1.5 points (*P* < 0.001) (A)). There was no significant difference in the ILD-GAPC score between with nonrespiratory mortality and without (3.0 ± 1.2 points vs. 2.2 ± 1.7 points (*P* = 0.098) (B)). Abbreviations: ILD, interstitial lung disease; GAPC, gender/age/physiology/Charlson Comorbidity Index score. Supplement Figure 2: Kaplan−Meier curves only in the %FVC >75% (%FVC score: 0 points) populations. Figure legends: in the %FVC >75% (%FVC score: 0 points) populations, ILD-GAPC better predicted the 3-year ILD-related events than ILD-GAP. Abbreviations: ILD, interstitial lung disease; G/A/P, gender/age/physiology; GAPC, gender/age/physiology/Charlson Comorbidity Index score. Supplement Figure 3: The Kaplan−Meier curves among patients with treatment interventions (*n* = 49). Figure legends: None of the Kaplan−Meier curves for predicting 3-year ILD-related events ((A) ILD-GAP model, (B) ILD-GAPC model) or 3-year all-cause mortality ((C) ILD-GAP model, and (D) ILD-GAPC model) showed significant differences. Abbreviations: ILD, interstitial lung disease; G/A/P, gender/age/physiology; GAPC, gender/age/physiology/Charlson Comorbidity Index score. Supplement Table 1: Relationships between ILD-GAP and ILD-GAPC. Footnote: The relationship between ILD-GAP and ILD-GAPC (*R* = 0.900 (*P* < 0.001)). Abbreviations: GAP, gender/age/physiology; GAPC, gender/age/physiology/Charlson Comorbidity Index score; ILD, interstitial lung disease.
