# Supplementary material for: ILD-GAP Combined with the Charlson Comorbidity Index Score (ILD-GAPC) as a Prognostic Prediction Model in Patients with Interstitial Lung Disease
Source: Can Respir J. 2023 Feb 8;2023:5088207. doi: 10.1155/2023/5088207 (PMC9931459; doi:10.1155/2023/5088207)
Supplement: Supplementary Materials — Supplement Figure 1: The ILD-GAPC score according to ILD-related events or nonrespiratory mortality. Figure legends: the ILD-GAPC score with ILD-related events was significantly higher than those without (3.6 ± 1.5 points (P < 0.001) (A)). There was no significant difference in the ILD-GAPC score between with nonrespiratory mortality and without (3.0 ± 1.2 points vs. 2.2 ± 1.7 points (P = 0.098) (B)). Abbreviations: ILD, interstitial lung disease; GAPC, gender/age/physiology/Charlson Comorbidity Index score. Supplement Figure 2: Kaplan−Meier curves only in the %FVC >75% (%FVC score: 0 points) populations. Figure legends: in the %FVC >75% (%FVC score: 0 points) populations, ILD-GAPC better predicted the 3-year ILD-related events than ILD-GAP. Abbreviations: ILD, interstitial lung disease; G/A/P, gender/age/physiology; GAPC, gender/age/physiology/Charlson Comorbidity Index score. Supplement Figure 3: The Kaplan−Meier curves among patients with treatment interventions (n = 49). Figure legends: None of the Kaplan−Meier curves for predicting 3-year ILD-related events ((A) ILD-GAP model, (B) ILD-GAPC model) or 3-year all-cause mortality ((C) ILD-GAP model, and (D) ILD-GAPC model) showed significant differences. Abbreviations: ILD, interstitial lung disease; G/A/P, gender/age/physiology; GAPC, gender/age/physiology/Charlson Comorbidity Index score. Supplement Table 1: Relationships between ILD-GAP and ILD-GAPC. Footnote: The relationship between ILD-GAP and ILD-GAPC (R = 0.900 (P < 0.001)). Abbreviations: GAP, gender/age/physiology; GAPC, gender/age/physiology/Charlson Comorbidity Index score; ILD, interstitial lung disease. [file 5088207.f1.docx]

**Supplement Figure 1. The ILD-GAPC score according to ILD-related events or non-respiratory mortality**

The ILD-GAPC score with ILD-related events was significantly higher than those without (3.6±1.5 points (P < 0.001) (A)). There was no significant difference in the ILD-GAPC score between with non-respiratory mortality and without (3.0±1.2 points vs. 2.2±1.7 points (P = 0.098) (B)).

**Abbreviations:** ILD, interstitial lung disease; GAPC, gender/age/physiology/Charlson Comorbidity Index score.**Supplement Figure 2. Kaplan-Meier curves only in the %FVC > 75% (%FVC score: 0 point) populations**

**
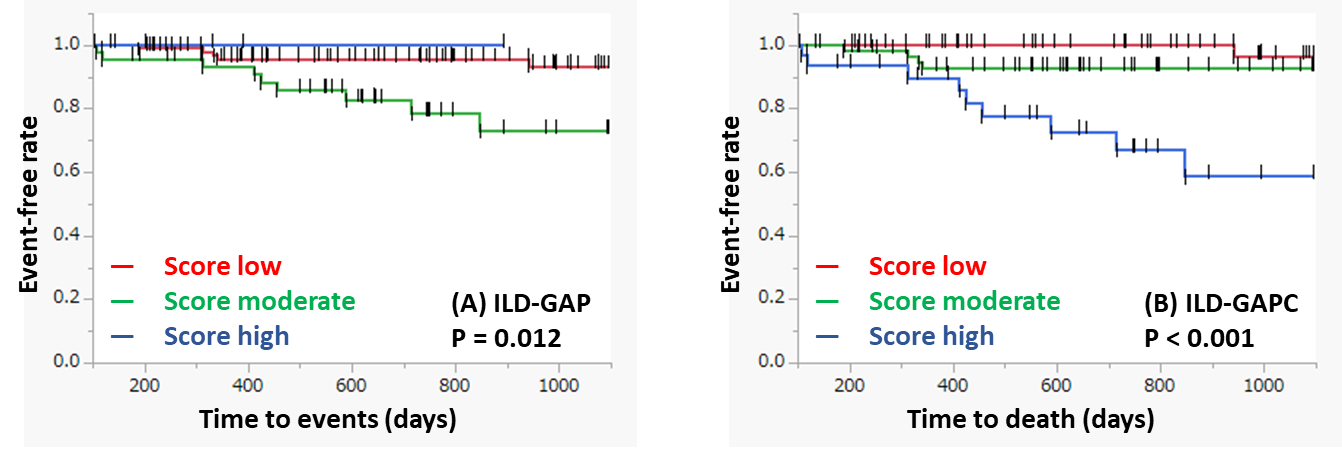
**

In the %FVC > 75% (%FVC score: 0 point) populations, ILD-GAPC better predicted the 3-year ILD related events than ILD-GAP. **Abbreviations:** ILD, interstitial lung disease; G/A/P, gender/age/physiology; GAPC, gender/age/physiology/Charlson Comorbidity Index score.

**Supplement Figure 3. The Kaplan-Meier curves among patients with treatment intervention (n = 49)**

None of the Kaplan-Meier curves for predicting 3-year ILD related events ((A) ILD-GAP model, (B) ILD-GAPC model) or 3-year all-cause mortality ((C) ILD-GAP model, and (D) ILD-GAPC model) were significant differences.

**Abbreviations:** ILD, interstitial lung disease; G/A/P, gender/age/physiology; GAPC, gender/age/physiology/Charlson Comorbidity Index score.

**Supplement Table 1. Relationships between ILD-GAP and ILD-GAPC**

|  | **ILD-GAP score low** | **ILD-GAP score moderate** | **ILD-GAP score high** | **Total** |
| --- | --- | --- | --- | --- |
| **ILD-GAPC score low** | **69** | **0** | **0** | **69** |
| **ILD-GAPC score moderate** | **48** | **25** | **0** | **73** |
| **ILD-GAPC score high** | **0** | **32** | **11** | **43** |
| **Total** | **117** | **57** | **11** | **185** |

**Footnote:**

The relationship between ILD-GAP and ILD-GAPC (R = 0.900 (P < 0.001)).

**Abbreviations:**

GAP, gender/age/physiology; GAPC, gender/age/physiology/Charlson Comorbidity Index score; ILD, interstitial lung disease.
